# Association between perceived fatigue and gait parameters measured by an instrumented treadmill in people with multiple sclerosis: a cross-sectional study

**DOI:** 10.1186/s12984-015-0028-2

**Published:** 2015-04-02

**Authors:** Alon Kalron

**Affiliations:** Department of Physical Therapy, Sackler Faculty of Medicine, Tel-Aviv University, Tel-Aviv, Israel; Multiple Sclerosis Center, Sheba Medical Center, Tel-Hashomer, Israel

**Keywords:** Multiple Sclerosis, Gait, Fatigue, Treadmill

## Abstract

**Background:**

Multiple sclerosis (MS) is a multi-focal progressive disorder of the central nervous system often resulting in diverse clinical manifestations. Symptomatic fatigue is quite common in people with MS (PwMS), with prevalence as high as 85%. Nevertheless, it remains poorly understood and its association with walking capabilities unclear. Therefore, the objective of this investigation was to examine the relationship between symptomatic fatigue and spatio-temporal parameters of gait in PwMS based on an instrumented treadmill.

**Methods:**

One hundred and twenty-four relapsing-remitting patients diagnosed with MS, 84 women and 40 men aged 42.6 (S.D = 11.9), participated in this investigation. A convenience sample of 25 apparently healthy subjects, 15 women and 10 men aged 40.3 (S.D = 11.1), served as controls. Gait spatiotemporal parameters were obtained using the Zebris FDM-T Treadmill (Zebris1 Medical GmbH, Germany). The Modified Fatigue Impact Scale (MFIS), a self-reported questionnaire, was used to determine the level of symptomatic fatigue in the MS study group. PwMS were divided into two groups: fatigued and non-fatigued.

**Results:**

Forty-four PwMS were classified as suffering from fatigue (mean MFIS = 52.0, S.D = 13.7); 80 were classified as non-fatigued (mean MFIS = 14.5, S.D = 14.5). Individuals in the fatigued group walked slower than those in the non-fatigued group; 1.7 (S.D = 2.4) vs. 2.4 (S.D = 1.0); P < 0.001, respectively. Moreover, fatigued patients took smaller steps, had a shorter stride length, prolonged stance, double support phase and a shorter single support phase compared to the non-fatigued group. In the total group, fatigue was significantly correlated with 10 (out of 14) spatiotemporal parameters of gait, however, correlation scores <0.40 were considered as weak correlations. According to step one of the linear logistic regression analysis, the temporal gait component was found to explain 5.1% of the variance related to symptomatic fatigue, R2 = 0.051, *χ*2 (1) = 6.511, P = 0.011. Step two of the model added the gait spatial component, thus increasing the explaining variance to 9.3%; R2 = 0.093, *χ*2 (2) = 12.12, P = 0.002. The asymmetry gait parameter did not contribute to the equation.

**Conclusions:**

Perceived fatigue is related to walking speed in PwMS, nevertheless its contribution to level of fatigue is limited.

## Background

Multiple sclerosis (MS) is a multi-focal progressive disorder of the central nervous system often resulting in diverse clinical manifestations. Symptomatic fatigue and walking capabilities are major challenges to the medical and rehabilitation management of persons with MS (PwMS). Both of these issues are quite common, with prevalence of fatigue as high as 85% [1] and impaired gait up to 75% [2]. Collectively, they are the main contributors to advanced disability and a poorer quality of life [3].

The etiology of symptomatic fatigue in PwMS is unclear, though likely multi-factorial. The most commonly proposed primary mechanisms of fatigue in MS encompass the immune system or damage to the central nervous system [4,5]. There is evidence that, in some instances, lesions in the basal ganglia, hypothalamus and parietal lobes may play an important role [6-9]. Diffuse demyelination and axonal lesions associated with reduced nerve conduction velocity and a prolonged refractory period have also been discussed as producing fatigue in MS [10,11]. Previous trials have reported a strong association between symptomatic fatigue with increased muscle fatigability [12] and impaired central muscle activation in PwMS [13]. Recently, symptomatic fatigue has been found to be associated with cerebellar and brainstem involvement [14].

Based on the knowledge that walking is a complex task requiring integration of many brain resources [15], we determined that it was necessary to examine the relationship between perceived fatigue and gait in PwMS. This was reinforced by researchers who found an association between elevated fatigue, impaired balance and a higher risk of falls in this population [16,17].

Various studies examining the association between gait performance and perceived fatigue have presented mixed and limited results. Huisinga et al. [18] found a correlation between fatigue measured by the Fatigue Severity Scale and deficits in ankle power generation at late stance, in mild disability MS participants (average EDSS = 2.6).In contrast, Noguiera et al. did not observe any interaction between the fatigue and ankle motion in 12 PwMS [19]. Sacco et al. [20] found a correlation between temporal-spatial parameters of gait (speed, cadence and stride length) collected by the GAITRite mat and fatigue (measured by the Wurzburg Fatigue Inventory for MS), revealing a significant negative correlation between velocity (r = -0.54), cadence (r = -0.44) and stride length (r = -0.5).

In cross-sectional studies, Motl et al. [21] and Sandroff et al. [22] reported similar findings when examining a relatively large group of PwMS assessed by the GAITRite mat and level of fatigue via the Fatigue Severity Scale. Nonetheless, Morris et al. [23] and Crenshaw et al. [24] found no correlation between fatigue and walking speed, step length and double support time even after induction of additional fatigue [25]. Thus, it seems that the evidence as to the association between gait and fatigue in PwMS, is still controversial.

An in-depth investigation clarifying the relationship between definite gait parameters to perceived fatigue may be facilitated in several ways. Firstly, management of fatigue- related symptoms may be improved. For the past several years, walking rehabilitation programs have been proposed as a legitimate treatment option aimed at reducing the level of perceived fatigue in PwMS [26]. Recently, Garret et al. demonstrated a reduction in symptomatic fatigue and an increase in walking speed following a 10-week community-based exercise program in 121 PwMS [27]. Secondly, quantified gait parameters may serve as markers denoting level of fatigue indicating a possibility that fatigued PwMS adopt a specific walking pattern. Recognition of this walking strategy may help clinicians identify fatigued MS patients. Finally, new information examining the relationship between gait impairments and fatigue may generate additional trials in order to examine the possibility of common central neural networks and resources. Therefore, the specific objective of this investigation was to examine the relationship between symptomatic fatigue and spatio-temporal parameters of gait, based on an instrumented treadmill in PwMS.

## Methods

### Study design and participants

This study was a cross sectional study and included a control group. One hundred and twenty-four relapsing-remitting patients diagnosed with MS, 84 women and 40 men aged 42.6 (S.D = 11.9), were recruited from the Multiple Sclerosis Center, Sheba Medical Center, Tel-Hashomer, Israel and participated in this investigation. Inclusion criteria for participants required: (1) a neurologist-confirmed diagnosis of definite relapsing-remitting MS according to the revised McDonald criteria [28]; (2) <6 on the Expanded Disability Status Scale (EDSS), equivalent to the ability to walk without an assistive device (e.g. a cane or walker); and (3) relapse-free for at least 30 days prior to testing. Exclusion criteria included: (1) orthopedic disorders that could negatively affect mobility; (2) no history of psychiatric problems that could negatively affect communication with the tester while walking on the treadmill; (3) pregnancy; (4) blurred vision; (5) cardiovascular disorders; (6) respiratory disorders; (7) or taking steroids or fampridine. A convenience sample of 25 apparently healthy subjects, 15 women and 10 men aged 40.3 (S.D = 11.1), served as controls. None of the healthy participants reported any medication intake or relevant health impairments (e.g. orthopedic, neurological, or internal diseases). The study was approved by the Sheba Institutional Review Board. All participating subjects signed an informed consent form.

### Gait assessment

Gait spatiotemporal parameters were obtained using the Zebris FDM-T Treadmill (Zebris1 Medical GmbH, Germany) fitted with an electronic mat of 10,240 miniature force sensors, each approximately 0.85 cm × 0.85 cm, embedded underneath the belt. The treadmill’s contact surface measures 150 cm x 50 cm and its speed can be adjusted from 0.2 and 22 km/h at intervals of 0.1 km/h. When the subject stands/walks on the treadmill, the force exerted by his feet (the so-called reactive-normal force in directions x, y and z) is recorded by the sensors at a sampling rate of 120 Hz. Due to the high density of the sensors, the foot is mapped at a high resolution to facilitate even subtle changes in force distribution. Timing can also be monitored. Dedicated software integrates the force signals and provides 2-D/3-D graphic representation of major spatiotemporal parameters during gait. Major spatio-temporal data included the following values: velocity (km/h), cadence (steps/min), stance phase (% gait cycle (%GC)), single and double support phases (%GC), width between steps (mm), step/stride length (cm) and step/stride time (s). Furthermore, step length differences and step time differences were calculated for each gait trial. These parameters were calculated as the absolute value of the differences between the corresponding right and left values.

In 2012, Faude et al. reported high levels of between- and within-day reliability in healthy seniors for the majority of spatiotemporal gait parameters recorded by the Zebris treadmill system during walking, with coefficients of variation typically below 5% and 7%, respectively [29].

### Symptomatic fatigue

The Modified Fatigue Impact Scale (MFIS), a self-reported questionnaire, was used to determine the level of symptomatic fatigue in the MS study group. The MFIS [14] is a multidimensional 21-item questionnaire capturing information as to the effects of fatigue within physical (9-items), psychosocial (2-items) and cognitive (10-items) domains over a period of four-weeks. Participants rated the 21 items on a 5-point Likert-type scale, ranging from never (0) to always (4). The MFIS yields three subscale scores and an overall score ranging from 0 to 84 (the higher scores indicate more fatigue). Previous studies have demonstrated that a total score of 38 is the cutoff for discriminating fatigued from non-fatigued individuals [30,31]. In addition to its multidimensional features, other advantages of the MFIS include easy to use, good reproducibility, and a strong correlation with results of the Fatigue Severity Scale (r = 0.68) [31].

### Experimental design

Upon acceptance to the study, participants were instructed to fill out the MFIS form. Prior to the treadmill gait measurement phase, all participants actively participated in an adaptation-familiarization trial in order to establish each individual’s speed level. Starting at a fixed speed of 0.5 km/h, belt speed was increased by 0.3km/h every 15 seconds, in a stepwise manner. When the participant informed the tester as to the speed that best characterized his/her normal walking pace, it was designated as his/her comfort speed. Following this adaptation phase that lasted approximately 2 minutes, a 30 second break was provided. Subsequently, each participant was instructed to walk barefoot on the treadmill for one consecutive minute at their comfort speed. The time period was selected in concurrence with a previous study demonstrating that gait data measured for one minute by the Zebris treadmill are valid markers of neurological impairment in PwMS [32]. Gait assessment was performed at the Center of Advanced Technologies in Rehabilitation, Sheba Medical Center. Measurements were calculated by an experienced physical therapist specialized in neurological rehabilitation.

### Statistical analysis

MS subjects were divided into two groups: fatigued and non-fatigued. Allocation was determined according to the MFIS scores. The cut-off point for distribution was set at 38. Pw MS with scores ≥38 were assigned to the fatigued group and those with <38 were assigned to the non-fatigued group. The selected cut-off point was based on Flachenecker et al’s [33] study that correlated the MFIS with another fatigue inventory and its defined scores for fatigued and non-fatigued. Group differences in age and gender distribution were determined using an independent sample-t and chi-square test, respectively. All spatio-temporal parameters of gait data were normally distributed according to the Kolmogorov-Smirnov test. Thus, differences in gait parameters between PwMS subgroups and controls were determined using the multivariate analysis of variance (MANOVA) test. The Bonferroni test enabled paired multiple comparisons between groups. The magnitudes of group differences were indexed by a 95% confidence interval (95% CI).

The association between symptomatic fatigue expressed by the MFIS questionnaire and spatio-temporal gait parameters were measured by Spearman’s rank-order correlation coefficient tests. Correlation coefficients which are ≤ 0.35 are generally considered to represent low or weak correlations, 0.36 to 0.67 modest or moderate correlations, and 0.68 to 1.0 strong or high correlations with r coefficients ≥ 0.90 very high correlations [34].

The evaluation of potential predictors for fatigue status (i.e., the dependent variable) was calculated by a stepwise linear regression analysis. Spatio-temporal parameters of gait of the overall MS sample were entered in to the model as covariates. At each subsequent step, the regression equations comprised those variables reaching specific thresholds of F and P values (for variable inclusion, F ≥ 1 and P ≤ 0.05; for exclusion, F < 1 and P > 0.05).

Importantly, in order to eliminate redundancy between correlated gait variables, a principal component analysis (PCA) was executed prior to the regression model. This procedure extracts variables that carry the most variance thus limiting a type I error. The PCA includes the Kaiser-Meyer-Olkin measure of sampling adequacy and Bartlett’s test of sphericity. Extraction of variables was based on eigenvalues >1.0. Oblimn with Kaiser normalization used as the rotation method.

All analyses were performed using IBM SPSS statistics software (Version 21.0 for Windows, SPSS Inc. NY, USA). All reported P-values were two-tailed. The level of significance was set at P < 0.05.

## Results

### Demographics

Forty-four PwMS (35.5%) were classified as suffering from fatigue (mean MFIS = 52.0, S.D = 13.7); 80 (64.5%) were classified as non-fatigued (mean MFIS = 14.5, S.D = 14.5). PwMS in the fatigued group had an elevated EDSS score compared to the non-fatigued group, 3.7 (S.D = 1.4) vs. 1.2 (S.D = 1.1); P < 0.001, respectively. As to gender, the fatigued group consisted of 68.2% women while the non-fatigued, 67.5% women. No differences were observed between the PwMS subgroups in terms of age, height, body mass and disease duration. The individuals’ characteristics and neurological assessment scores are summarized in Table 1.

**Table 1:** **Demographic, anthropometric, clinical characteristics of the study population**

**Variables**	**Mean (S.D.)**			**P- value between MS subgroups**
	**Patients without fatigue (n = 80)**	**Patients with fatigue (n = 44)**	**Healthy subjects (n = 25)**	
Age (years)	40.9 (15.5)	44.2 (12.7)	40.3 (11.1)	0.465
Gender				
Female	54	30	15	
Male	26	14	10	
Disease duration (years)	7.0 (8.4)	8.1 (7.8)	---	0.469
Height (cm)	169.4 (9.6)	167.8 (7.2)	168.7 (8.5)	0.364
Body mass (kg)	69.0 (15.9)	67.8 (12.0)	68.3 (14.6)	0.641
MFIS (range 0-84)	14.5 (14.5)	52.0 (13.7)	---	<0.001
EDSS	2.5 (1.6)	3.7 (1.8)	---	<0.001
Pyramidal	1.6 (1.2)	2.1 (1.2)	---	0.013
Cerebellar	0.9 (1.0)	1.3 (1.2)	---	0.08
Sensory	0.7 (0.9)	1.2 (1.1)	---	0.01
Brainstem	0.5 (0.2)	0.4 (0.7)	---	0.571
Visual	0.2 (0.1)	0.2 (0.4)	---	0.764
Cerebral	0.2 (0.5)	0.0 (0.2)	---	0.100

EDSS: expanded disability status scale, MFIS: Modified fatigue impact scale.

### Spatio-temporal gait findings

#### Fatigued vs. Non-fatigued

The mean gait velocity obtained from the instrumented treadmill for the study sample, was within the range for usual gait speed among PwMS [35]. Individuals in the fatigued group walked slower than participants in the non-fatigued group; 1.7 (S.D = 2.4) vs. 2.4 (S.D = 1.0) (km/h), P < 0.001, respectively. Moreover, fatigued patients took smaller steps, had a shorter stride length, prolonged stance, double support phase and a shorter single support phase compared to the non-fatigued group. Additionally, the fatigued group exhibited a larger asymmetry between the right and left legs in 3 (out of 4) parameters; step length, single support and stance phase compared to the non-fatigued group. No differences between groups were observed in terms of step width and step time asymmetry. Gait variables in the subgroups of PwMS are provided in Table 2.

**Table 2:** **Spatio-temporal gait parameters of study participants**

**Gait variable**	**Mean (S.D.)**			**F,** ***P*** **-Value**	**Mean difference (95% CI)** ***P-*** **Value**		
	**Non-fatigued (n = 80)**	**Fatigue (n = 44)**	**Healthy subjects (n = 25)**		**Non fatigue - fatigue**	**Fatigue - Healthy**	**Non fatigue - Healthy**
Velocity (km/h)	2.4 (1.0)	1.7 (1.0)	3.5 (0.7)	25.4 (<0.001)	0.7 (0.2, 1.1) P = 0.001*	−1.8 (-2.4, -1.2) P < 0.001*	−1.1 (-1.7, -0.6) P < 0.001*
Cadence (steps/min)	96.1 (17.2)	86.2 (21.4)	100.6 (11.7)	6.3 (0.002)	9.9 (1.7, 18.0) P = 0.012*	−14.4 (-25.5, -3.3) P = 0.006*	−4.5 (-14.8, 5.7) P = 0.855
Mean Step time (sec)	0.65 (0.14)	0.78 (0.40)	0.60 (0.07)	5.4 (0.005)	−0.13 (-0.24, -0.02) P = 0.015*	0.18 (0.02, 0.33) P = 0.018*	0.05 (-0.10, 0.18) P = 1.00
Mean Step length (sec)	40.2 (14.5)	32.0 (14.9)	58.1 (8.5)	26.7 (<0.001)	8.1 (1.8, 14.4) P = 0.07*	−26.1 (-34.7, -17.4) P < 0.001*	−17.9 (-25.9, -1.0) P < 0.001*
Mean Stance (%GC)	68.0 (5.2)	71.9 (6.5)	64.3 (2.1)	16.5 (<0.001)	−3.9 (-6.3, -1.5) P < 0.001*	7.6 (4.3, 10.9) P < 0.001*	3.7 (0.6, 6.7) P = 0.012*
Mean Single support (% GC)	32.0 (5.2)	28.1 (6.5)	35.7 (2.1)	16.4 (<0.001)	3.9 (1.5, 6.3) P < 0.001*	−7.6 (-10.9, -4.3) P < 0.001*	−3.7 (-6.7, -0.7) P = 0.011*
Total Double support (% GC)	36.1 (10.5)	43.9 (13.1)	28.6 (4.1)	16.5 (<0.001)	−7.8 (-12.7, -3.0) P < 0.001*	15.2 (8.6, 21.9) P < 0.001*	7.4 (1.3, 13.5) P = 0.012*
Mean Step width (cm)	13.6 (4.5)	14.7 (3.7)	11.1 (3.1)	5.8 (0.004)	−1.1 (-3.0, 0.7) P = 0.430	3.6 (1.0, 6.1) P = 0.002*	2.4 (0.1,4.8) P = 0.036*
Mean Stride time (sec)	1.30 (0.28)	1.56 (0.80)	1.21 (0.15)	5.4 (0.005)	−0.26 (-0.48, -0.04) P = 0.015*	0.35 (0.05, 0.65) P = 0.018*	0.09 (-0.19, 0.37) P = 1.00
Mean Stride length (cm)	80.3 (29.0)	64.0 (29.8)	116.2 (17.0)	26.7 (<0.001)	16.3 (3.6, 28.7) P = 0.007*	−52.1 (69.4, -34.9) P < 0.001*	−35.9 (-51.8, -20.0) P < 0.001*
Step length asymmetry (cm)	2.7 (2.40)	3.6 (3.28)	1.8 (1.32)	3.8 (0.025)	−0.9 (-2.0, 0.3) P = 0.237	1.8 (0.18, 3.4) P = 0.024*	0.93 (-0.5, 2.4) P = 0.387
Step time asymmetry (msec)	0.03 (0.05)	0.07 (0.07)	0.01 (0.01)	10.8 (<0.001)	−0.03 (-0.06, -0.008) P = 0.004*	0.06 (0.027, 0.091) P < 0.001*	0.03 (-0.002, 0.057) P = 0.08
Single support asymmetry (GC%)	2.2 (2.1)	2.6 (2.6)	0.7 (0.7)	6.1 (0.003)	−0.40 (-1.4, 0.6) P = 0.983	1.9 (0.5, 3.2) P = 0.003*	1.5 (0.3, 2.7) P = 0.012*
Stance asymmetry (GC%)	2.2 (2.1)	2.6 (2.7)	0.7 (0.70	6.1 (0.003)	−0.4 (-1.4, 0.6) P = 0.921	1.9 (0.6, 3.3) P = 0.002*	1.5 (0.3, 2.7) P = 0.012*

*P<0.05.

#### Healthy vs. non-fatigued MS patients

Step time of the non-fatigued patients was significantly slower compared to the healthy participants, 2.4 (S.D = 1.0) vs. 3.5 (S.D = 0.7) (km/h), P < 0.001, respectively. Additionally, differences were observed in gait parameters related to spatial measurements. Patients walked with a smaller step and stride length and a wider base of support. No differences between groups were demonstrated in step time, stride time and cadence. Non-fatigued patients demonstrated a larger asymmetry between the right and left legs in the context of single support and stance phase. In contrast, there were no differences in step length and time associated with asymmetry. Gait variables in PwMS and healthy subjects are provided in Table 2.

#### Healthy vs. fatigued MS patients

Our findings correspond to those reported in the previous section. Fatigued patients walked slower with smaller steps and stride length, lower cadence, prolonged stance and double support phase, wider base of support and a shorter single support phase compared to healthy controls. In addition, fatigued patients demonstrated elevated asymmetry between legs in terms of step length, step time, single support and stance phase compared to their healthy counterparts.

### Correlation between gait measurements and symptomatic fatigue

In the entire group, fatigue was significantly correlated with 10 (out of 14) spatiotemporal parameters of gait. However, correlation scores <0.40 were considered weak correlations. Parameters not found to be significantly correlated to the MFIS score were step width and 3 (out of 4) asymmetry scores; step length, single support and stance. In the non-fatigued group, correlations were observed in 7 (out of 14) gait parameters. The strongest scores were found for stance, single support and double support phases, 0.356, -0.357, 0.359, respectively. In contrast, no significant correlations were found between the MFIS score and gait parameters in the fatigued group. Additionally, with respect to PwMS subgroups, no correlations were found between the MFIS score to all 4 gait asymmetry parameters. Correlation scores between gait measurements and symptomatic fatigue are detailed in Table 3.

**Table 3:** **Spearman’s rho correlation scores;** ***P*** **-Value, between symptomatic fatigue (MFIS) to gait parameters**

**Gait variable**	**Total group (n = 124)**	**Fatigue (n = 44)**	**Non-fatigue (n = 80)**
Velocity (km/h)	−0.339; P < 0.001*	0.021; P = 0.890	−0.237; P = 0.034*
Cadence (steps/min)	−0.322; P < 0.001*	−0.143; P = 0.356	−0.285; P = 0.010*
Mean Step time (sec)	0.328; P < 0.001*	0.143; P = 0.356	0.284; P = 0.011*
Mean Step length (sec)	−0.274; P = 0.002*	0.037; P = 0.810	−0.172; P = 0.128
Mean Stance (%GC)	0.372; P < 0.001*	−0.071; P = 0.648	0.356; P = 0.001*
Mean Single support (% GC)	−0.372; P < 0.001*	0.086; P = 0.579	−0.357; P = 0.001*
Total Double support (% GC)	0.373; P < 0.001*	−0.077; P = 0.621	0.359; P = 0.001*
Mean Step width (cm)	0.171; P = 0.057	0.002; P = 0.988	0.063; P = 0.579
Mean Stride time (sec)	0.325; P < 0.001*	0.137; P = 0.374	0.285; P = 0.011*
Mean Stride length (cm)	−0.274; P = 0.002*	0.037; P = 0.809	−0.172; P = 0.128
Step length asymmetry (cm)	0.094; P = 0.299	−0.269; P = 0.077	0.167; P = 0.139
Step time asymmetry (msec)	0.205; P = 0.022*	−0.155; P = 0.316	0.057; P = 0.618
Single support asymmetry (GC%)	0.052; P = 0.569	−0.030; P = 0.846	−0.007; P = 0.947
Stance asymmetry (GC%)	0.059; P = 0.515	−0.042; P = 0.789	0.025; P = 0.829

*P < 0.05.

### Principal component analysis and linear regression analysis

Bartlett’s test was significant (P < 0.001) indicating appropriateness of the PCA for the present data set of spatio-temporal gait variables. Three components had eigenvalues >1.0 indicating eligibility for logistic regression analysis. Eigenvalues of the three components presented in a descending order were 7.86, 1.91, and 1.85, respectively. Additionally, the cumulative % for the three components was 56.3%, 13.7% and 13.2%, respectively, (a total cumulative % of the selected components was 83.0%). Gait variables highly loaded with the first component included, in particular, spatial parameters: mean step length and mean stride length (referred to as the spatial gait component). Variables highly loaded with the second component included temporal gait parameters: stride time, step time and cadence (referred to as the temporal gait component). The third component was highly loaded with gait asymmetrical variables: single support asymmetry, stance phase asymmetry and step time asymmetry (referred to as the asymmetry gait component).

On basis of the PCA findings, a linear regression analysis was conducted. The three components derived from the PCA were entered into the analysis as independent variables; the MFIS score was defined as the dependent variable. The results of the regression model are shown in Table 4.

**Table 4:** **Linear regression analysis for symptomatic fatigue according to spatio-temporal gait component**

**Model**	**Variables**	**Beta (Std.Error)**	**t,** ***P*** **-value**	**Odds ratio (95% C.I.)**
1	Temporal gait component	0.538 (0.245)	4.805, P = 0.028*	1.713 (1.059, 2.771)
	Constant	−0.613(0.193)	10.113, P = 0.001*	0.542
2	Spatial gait component	−0.482 (0.207)	5.414, P = 0.020*	0.617 (0.411, 0.927)
	Temporal gait component	0.476 (0.219)	4.717, P = 0.030*	1.609 (1.048, 2.472)
	Constant	−0.737 (0.207)	12.625, P < 0.001*	1.3, 5.1

*P < 0.05.

According to step one of the model, the temporal gait component was found to explain 5.1% of the variance related to symptomatic fatigue, as reported by the MFIS self-reported questionnaire, R2 = 0.051, *χ*2 (1) = 6.511, P = 0.011. Step two of the model added the gait spatial component, thus increasing the explaining variance to 9.3%; R2 = 0.093, *χ*2 (2) = 12.12, P = 0.002. The asymmetry gait parameter did not explain the variance related to symptomatic fatigue.

## Discussion

The primary objective of the present study was to examine the relationship between perceived fatigue and spatio-temporal parameters of gait measured by an instrumented treadmill in PwMS. Given the high prevalence of these symptoms in the MS population in addition to conflicting evidence as to the correlation between the two, supported this aim. Moreover, clarification of this issue can be helpful to PwMS who suffer from one or both of these symptoms and their corresponding medical staff who often encounter these complaints. Moreover, we examined gait with a relatively innovative instrument. Compared to standard electronic walkways, this device enables measurement over longer distances, believed to be relevant for a relatively younger aged experimental group.

Compared to the non-fatigued participants, patients in the fatigued group walked significantly slower, with shorter steps, a longer step time and prolonged double support period. In total, the fatigued group performed poorer in 9 (out of 14) gait parameters compared to the non-fatigued participants. Moreover, according to the correlation analysis, perceived fatigue was correlated with 10 (out of 14) gait parameters in the total sample group. The strongest correlations were found for velocity (-0.339), single (-0.372) and double support period (0.373).

Our findings concur with Sacco et al’s [20] and Motl et al’s. [21] studies. Sacco showed negative correlations between level of fatigue (physical sub category) and velocity (-0.54), cadence (-0.44) and stride length (-0.50). Similarly, Motl et al. reported significant correlations between symptomatic fatigue and gait speed (-0.324), stride length (-0.372) and double support period (0.420).

Interestingly, significant correlations between gait parameters to the level of self-reported fatigue were found solely in the non-fatigue group. No correlations were observed in the MS fatigued patients. A possible explanation could be a floor effect in terms of walking speed. PwMS in the fatigue group walked at a mean speed of 1.7 (km/h) which is considered very slow, however, relatively safe. Reducing speed at this rate is nearly impossible since the walking belt of the treadmill barely moves. There is a logical possibility that the majority of people in the fatigue group preferred to ambulate within a safe walking speed, regardless of the level of fatigue, consequently, eliminating the likelihood of a significant correlation between gait speed and perceived fatigue. This trend did not occur in the non-fatigued participants’ group, who walked at a speed of 3.7 (km/h). In this case, patients had a better possibility to adjust to the speed of the belt according to their comfort level.

We are aware that one may argue that the different gait performances observed between groups was simply a reflection of the general neurological condition represented by the EDSS score. The mean EDSS score of the fatigued patients were higher compared to the non-fatigued participants; 3.7 (S.D = 1.8) and 2.5 (S.D = 1.6), P < 0.001, respectively. Therefore, in order to address this assertion, a regression model for the entire MS group was performed. The MFIS was selected as the dependent variable while spatio-temporal gait parameters served as explaining variables. Accordingly, we found that the temporal component of gait (mainly indicating walking speed) explained only 5.4% of the variance related to symptomatic fatigue; hence, the majority of the variance should be explained by other factors. Therefore, our results preclude the ability to draw a firm conclusion as to the relationship between definite gait parameters and perceived fatigue.

However, we present several possibilities that could be considered a link between gait impairment and MS related fatigue. Morgante et al. conducted an MRI and electrophysiological study on PwMS [36]. Their main findings demonstrated that an increased burden of lesion load in frontal areas correlates with the degree of fatigue itself, expressed by the FSS score. This was paralleled by a functional impairment of the motor areas involved in movement planning and preparation. Interestingly, disturbances in the frontal lobe were found related to a cautious and slower gait pattern in the elderly [37]. Consequently, we raise the possibility that lesions in the frontal lobe can be attributed to both MS related fatigue and a slower walking pattern.

To prove this hypothesis, we suggest that future studies add a dual-task walking test to the common gait measurements. A dual walking-thinking task requires additional frontal lobe sources compared to normal walking, thus providing an opportunity to precisely examine the relationship between fatigue, gait and frontal lobe activation in the MS population.

Likewise, energy cost may link perceived fatigue and walking disturbances in PwMS. However, this issue is still controversial. Motl et al. [21] reported that energy cost was inversely associated with gait speed and perceived fatigue as measured by FSS in people with mild MS. In contrast, Kempen et al. [38] claimed that perceived fatigue and energy cost during walking are not associated with mild to moderate walking problems, indicating that these are two separate constructs. Future studies are warranted to clarify this debate.

Several clinical implications may be drawn from our data. Practitioners should be aware that perceived fatigue could contribute to a slower walking speed in PwMS. Therefore, we propose that the management strategy to reduce fatigue should include a walking intervention program. Specifying that a treatment program designed to increase walking speed may reduce the level of MS related fatigue.

On the other hand, in order to increase PwMS’s walking speed, a rehabilitation program based on a behavioral component such as cognitive behavioral therapy may be effective.

Interestingly, Bol et al. compared a cognitive-behavioral approach with a biomechanical approach to explain fatigue and physical disability in MS patients. Neither the cognitive-behavioral nor the biomechanical model was adequate. However, by incorporating the two models suggests that an integrated approach could be beneficial in the treatment of fatigue and gait [39]. Similarly, Coote et al published a study protocol entitled “step it up” directed at managing major symptoms of PwMS [40]. The protocol describes a rehabilitation program based on 10 weeks of exercise plus a social cognitive theory based on behavioral change intervention. The authors plan to examine the efficacy of this program on major MS-related symptoms, including perceived fatigue.

Our study has several limitations. Firstly, categorization between fatigued and non-fatigued PwMS was based solely on the 38 point MFIS cut-off point. Although Flachenecker et al’s [33] study reported that this score is appropriate to differentiate between fatigue to non-fatigued PwMS, a possibility exists that several participants were incorrectly classified, especially in cases where the MFIS score was relatively close to the cut-off point. Secondly, although this device is commonly used in the general population, walking on a treadmill does not duplicate walking in the natural environment. The main differences consist of the afferent impulse and fixed speed. These distinctions may have partly contributed to the differences demonstrated between the groups. Nevertheless, walking speed in the present MS study group was similar with those reported by different trials measuring walking velocity on electronic mats in the MS population.

We further note that we did not take into account additional factors related to symptomatic fatigue such as immunological abnormalities, depression and sleep disorders, although the exact role of these variables in MS related fatigue, is not fully understood. Furthermore, the addition of physiological measurements during walking such as heart rate and/or expired air measures of VO_2_ may have facilitated substantial information related to level of fatigue.

Finally, the cross-sectional study design is a limitation because it does not provide longitudinal data on the possibility that elevated fatigue results in a worsening of community ambulation and fatigue over time in PwMS. This design does not identify the direction of causality among perceived fatigue and gait parameters; however, cross-sectional data are generally a precursor for the design of randomized controlled trials that isolate causality.

## Conclusions

Presently, an important debate exists as to how MS-related fatigue relates to the origin of the fatigue, since this may determine the most appropriate treatment. Our study demonstrated that definite gait parameters are associated with perceived fatigue in persons with MS. Clinicians should be aware that PwMS who suffer from perceived fatigue, walk slower, with shorter steps, a longer step time and prolonged double support period. Therefore, we propose that the management strategy to reduce fatigue should include a walking intervention program. Alternatively, in order to increase PwMS’s walking speed, a rehabilitation program based on a behavioral component may be effective.

## Abbreviations

MS: Multiple sclerosis
PwMS: Persons with MS
EDSS: Expanded Disability Status Scale
CoP: Center of pressure
MFIS: Modified Fatigue Impact Scale
MANOVA: Multivariate analysis of variance
PCA: Principal component analysis
